# Major Bleeding Complications in COVID-19 Patients

**DOI:** 10.7759/cureus.16816

**Published:** 2021-08-01

**Authors:** Ignacio Boira, Violeta Esteban, Sandra Vañes, Carmen Castelló, Carly Celis, Eusebi Chiner

**Affiliations:** 1 Pulmonology, Hospital Universitario San Juan Alicante, San Juan de Alicante, ESP

**Keywords:** bleeding, covid-19, anticoagulation, hematoma, embolization

## Abstract

Severe acute respiratory syndrome coronavirus 2 (SARS-CoV-2) infection has been associated with thrombotic phenomena in the early stages of the disease, but also, less frequently, with major bleeding between the second and third week after onset, particularly in patients treated with therapeutic anticoagulation. This article describes four cases of patients admitted to the hospital with severe SARS-CoV-2 pneumonia who had arterial bleeding as a complication while on low-molecular-weight heparin at therapeutic doses. Half of the patients were women.

## Introduction

The disease resulting from severe acute respiratory syndrome coronavirus 2 (SARS-CoV-2) infection (coronavirus disease 2019, COVID-19) has constituted an unprecedented health problem worldwide, with a considerable economic, social, and health impact. In Spain, respiratory distress was reported in 31.1% of a multicenter cohort comprising more than 15,000 hospitalized patients, with a mortality rate of 30.7% in those aged 70 years and older [1]. COVID-19 has a wide clinical spectrum and clinicians must look out for rare but potentially serious manifestations such as hemorrhage, even if the published evidence is limited.

Here we describe four cases of major bleeding in COVID-19 patients. These events were severe and difficult to control, and all bar one was resolved through embolization by an interventional radiologist. Through these cases, we will review the mechanisms associated with SARS-CoV-2 pneumonia that increase the risk of arterial bleeding, namely microvascular damage and anticoagulation treatment.

## Case presentation

Case 1

**Figure 1:**
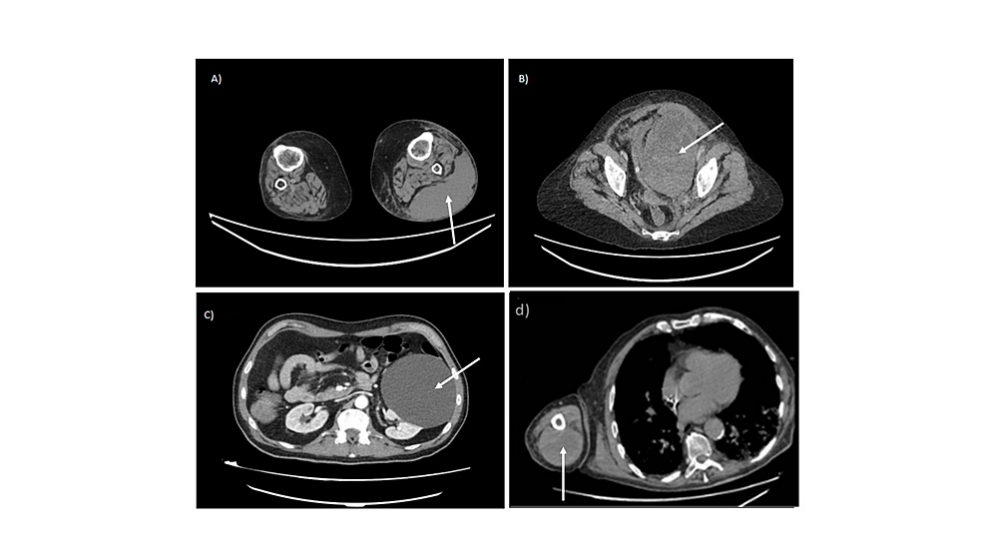
A) Extramuscular hematoma in the left anterior tibial region. B) Hematoma in the pelvis and in the lower third of the rectus abdominis. C) Hematoma in the lower rectus abdominis with signs of active bleeding. D) Hematoma in the posterior region of the right arm, in the location of the triceps, with active bleeding.

A 68-year-old woman with a history of metabolic syndrome was admitted to the ICU for 18 days with type 1 acute respiratory failure (ARF) secondary to bilateral SARS-CoV-2 pneumonia. She was transferred to the pulmonology ward when her condition improved. A sudden episode of dyspnea accompanied by elevated levels of D-dimer (4780 ng/mL) prompted a CT scan, which showed peripheral pulmonary thromboembolism. Her platelet count was 230,000/mcL. We, therefore, prescribed anticoagulation with enoxaparin (80 mg/12 h). After 72 h, the patient experienced intense pain due to a spontaneous hematoma in the left lower limb. An emergency vascular CT scan of the lower limbs (Figure 1A) showed a large hematoma (11 cm x 8 cm x 30 cm) in the left anterior tibial region, with a mass effect and active arterial bleeding causing compartment syndrome. Then, anticoagulation was discontinued and the hematoma was treated with embolization and subsequent surgical drainage.

Case 2

A 60-year-old woman with no relevant medical history was admitted with bilateral SARS-CoV-2 pneumonia and was later transferred to the ICU with ARF that was unresponsive to oxygen therapy. There she was intubated and put on invasive mechanical ventilation. Her condition improved, and on day 10 after admission, she was transferred to the pulmonology ward. At this point, she was on enoxaparin (60 mg/12 h) owing to an elevated D-dimer concentration (3339 ng/mL). Her platelet count was 334,000/mcL. Anuria and severe anemia motivated an emergency CT scan of the abdomen and pelvis (Figure 1B) after two days on the ward. This examination showed an extraperitoneal hematoma in the pelvis and another hematoma in the lower third of the left rectus abdominis, with signs of active arterial bleeding. After this finding anticoagulation was withdrawn and the hematoma was treated with embolization.

Case 3

A 66-year-old man with no relevant medical history was admitted with bilateral SARS-CoV-2 pneumonia and type 1 ARF. He was started on enoxaparin (60 mg/12 h) after laboratory tests detected a D-dimer concentration of 2850 ng/mL. He had mild thrombocytopenia with a platelet count of 123,000/mcL. On day 14 after admission, he experienced intense hypogastric pain associated with hypotension and tachycardia. Physical examination revealed a mass in the suprapubic area. An emergency blood test showed a hemoglobin level of 9.2 g/dL (five points below the last recorded level). An emergency CT scan of the abdomen and pelvis (Figure 1C) showed a hematoma in the lower rectus abdominis with active arterial bleeding and another hematoma in the pelvis. In view of these findings, anticoagulation was discontinued and embolization was performed. 

Case 4

An 87-year-old man, with a history of severe chronic obstructive pulmonary disease and chronic respiratory failure treated with 24-h home oxygen therapy at 2 L/min, was admitted with bilateral SARS-CoV-2 pneumonia and severe respiratory failure that required him to stay in a high-flow oxygen therapy ward. We put him on enoxaparin (60 mg/12 h) after detecting a D-dimer level of 2540 ng/mL, and on day 7 of his stay, he presented a tense hematoma in the right arm causing compartment syndrome. She had mild thrombocytopenia with a platelet count of 137,000/mcL. An emergency vascular CT scan (Figure 1D) showed a hematoma in the posterior region of the right arm, in the location of the triceps, with active bleeding. The patient was taken off the anticoagulant and underwent emergency fasciotomy and surgical drainage of the hematoma. A compression bandage was applied, and 48 h later the wound was closed. 

## Discussion

SARS-CoV-2 infection greatly affects hematopoiesis and vascular homeostasis, and it can bring about coagulation cascade activation, fibrinolysis, and, in severe cases, disseminated intravascular coagulation. This latter complication, unlike sepsis, is not linked to thrombocytopenia and involves a lower consumption of coagulation factors. In this process, plasminogen is converted to plasmin, which is responsible for breaking down fibrin, giving rise to degradation products such as D-dimer.

SARS-CoV-2 binds to angiotensin-converting enzyme 2 (ACE-2) receptors, causing a decrease in their activity. This triggers an immune response and activates the renin-angiotensin-aldosterone axis, causing an increase in blood pressure and thus favoring endothelial dysfunction and bleeding [2].

In a systematic review of 58 studies on COVID-19, thrombocytopenia was detected in 22.9% of the 6892 included patients and elevated D-dimer levels in 34.6% [3]. Both parameters were associated with greater severity, with the strongest association found for elevated D-dimer (OR 4.03) [3], a known prognostic factor for thrombosis, ventilatory support, and mortality.

COVID-19 is related to thrombotic phenomena. Recent studies have shown that patients with ARF treated in the ICU are at 15%-30% greater risk of venous thromboembolism, and those receiving conventional inpatient care have a 7% increased risk [4]. For this reason, clinical practice guidelines recommend thromboprophylaxis with heparin in all patients hospitalized for COVID-19. Heparin exerts an anti-inflammatory effect by reducing IL-6, and an antiviral effect by binding to the surface protein S1 and to the heparan sulfate proteoglycans present in ACE-2 receptors, preventing contact and fusion with the membrane [4].

According to most publications to date, there is no indication for empirical full-dose anticoagulation in patients with COVID-19 disease, unless clinical thrombosis or thromboembolism has been documented or there is another classic indication for its use (mechanical prosthetic valve, atrial fibrillation, etc.). In fact, to date, there is no published evidence to justify increasing the dose of heparin in patients with severe COVID-19, so it should only be used in the context of a controlled clinical trial.

There are very few published descriptions of bleeding events in COVID-19 patients. In a study by Pavlov et al. [5], 18% of patients admitted to the ICU with COVID-19 had a major bleeding episode. Major bleeding is defined as any bleeding that is fatal, that occurs in a critical organ (e.g. intracranial, intraspinal, intraocular, retroperitoneal, intra-articular, pericardial, or intramuscular with compartment syndrome), that causes hemoglobin levels to drop by 2 g/dL or more, or that requires transfusion of two or more units of packed red blood cells [6].

The most common bleeding complication is cerebral hemorrhage, which frequently affects older people with thrombocytopenia. The incidence of a cerebral hemorrhage in people with COVID-19 is 0.6 cases per 100 patients and the mortality rate is 45.7%, which shows this is a rare but clinically significant event [7].

Another complication is adrenal insufficiency secondary to bleeding. The mechanism of this condition is increased blood flow and reduced venous return, resulting in thrombosis and hemorrhage. The literature also contains cases of gastrointestinal bleeding [8] and retinal bleeding.

Hemorrhage in soft tissue, muscle, and the retroperitoneal space has been poorly described but is associated with high mortality. The arteries most commonly involved are the femoral artery, epigastric artery, and superior thoracic artery. A causal and temporal relationship has been found with SARS-CoV-2 infection; hence it is crucial to look out for this bleeding complication in COVID-19 patients on heparin or with trauma [9].

**Figure 2:**
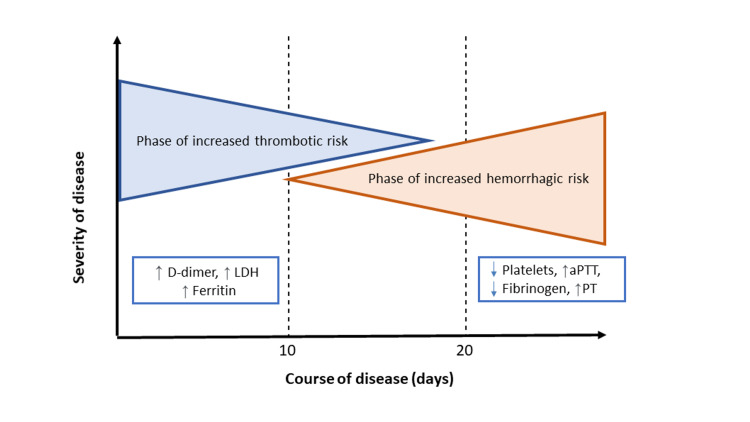
Biphasic course of thrombotic risk – bleeding in COVID-19 patients. PT, prothrombin time; aPTT, activated partial thromboplastin time; LDH, lactate dehydrogenase

Major bleeding generally occurs between the second and third week after admission, while thrombotic phenomena are more frequent in the first week. After the hyperinflammatory phase, D-dimer and fibrinogen levels fall, which can strengthen the anticoagulant effect and lead to overdose (Figure 2). In a study of 56 patients who were admitted to the ICU with COVID-19 and who suffered major bleeding, the investigators found that fibrinogen levels fell three to five days before the bleeding occurred. This could be a useful marker, alongside anti-factor Xa activity, for optimizing heparin dosing [10].

Thromboprophylaxis must be adapted to the biphasic course of the infection, with an individualized assessment of bleeding risk for each patient. After the inflammatory phase and the period of increased thrombotic risk, if we observe decreased D-dimer and fibrinogen levels, we should consider reducing the dose of anticoagulant to a prophylactic level to prevent bleeding events.

## Conclusions

Patients with SARS-CoV-2 pneumonia are at greater risk of arterial bleeding not only because of the microvascular damage associated with the infection but also because of the anticoagulation treatment, especially at therapeutic doses. Bleeding risk must, therefore, be evaluated on a case-by-case basis. Fibrinogen may be a useful biomarker for early detection of bleeding risk.
